# Ultrasound Imaging of the Abdominal Wall and Trunk Muscles in Patients with Achilles Tendinopathy versus Healthy Participants

**DOI:** 10.3390/diagnostics10010017

**Published:** 2019-12-30

**Authors:** Carlos Romero-Morales, Pedro Martín-Llantino, César Calvo-Lobo, Marta San-Antolín, Daniel López-López, María Blanco-Morales, David Rodríguez-Sanz

**Affiliations:** 1 Faculty of Sport Sciences, Universidad Europea de Madrid, Villaviciosa de Odón, 28670 Madrid, Spain; carlosmorales92@hotmail.com (C.R.-M.); pejamalla@gmail.com (P.M.-L.); maria.blanco@universidadeuropea.es (M.B.-M.); 2 Facultad de Enfermería, Fisioterapia y Podología, Universidad Complutense de Madrid, 28040 Madrid, Spain; cescalvo@ucm.es (C.C.-L.); davidrodriguezsanz@gmail.com (D.R.-S.); 3 Department of Psychology, Universidad Europea de Madrid, Villaviciosa de Odón, 28670 Madrid, Spain; martasanantolin@gmail.com; 4 Research, Health and Podiatry Group, Department of Health Sciences, Faculty of Nursing and Podiatry, Universidade da Coruña, 15403 Ferrol, Spain

**Keywords:** Achilles tendinopathy, musculoskeletal disorders, ultrasonography

## Abstract

Purpose: To compare and quantify with ultrasound imaging (USI) the inter-recti distance (IRD), rectus abdominis (RA), external oblique (EO), internal oblique (IO), transversus abdominis (TrAb), and multifidus thickness and the RA and multifidus cross-sectional area (CSA) between individuals with and without chronic mid-portion Achilles tendinopathy (AT). Methods: A cross-sectional study. A sample of 143 patients were recruited and divided into two groups: A group comprised of chronic mid-portion AT (*n* = 71) and B group composed of healthy subjects (*n* = 72). The IRD, RA, EO, IO, TrAb, and multifidus thickness, as well as RA and multifidus CSA, were measured by USI. Results: USI measurements for the EO (*p* = 0.001), IO (*p* = 0.001), TrAb (*p* = 0.041) and RA (*p* = 0.001) thickness were decreased as well as IRD (*p* = 0.001) and multifidus thickness (*p* = 0.001) and CSA (*p* = 0.001) were increased for the tendinopathy group with respect the healthy group. Linear regression prediction models (*R*^2^ = 0.260 − 0.494; *p* < 0.05) for the IRD, RA, EO, and IO thickness (*R*^2^ = 0.494), as well as multifidus CSA and thickness were determined by weight, height, BMI and AT presence. Conclusions: EO, IO, TrAb, and RA thickness was reduced and IRD, multifidus thickness and CSA were increased in patients with AT.

## 1. Introduction

Chronic mid-portion Achilles tendinopathy (AT) is a very common overuse disease, and one of the most prevalent conditions in athletes and the general population [1]. In addition, Albers et al. [2] showed an AT incidence rate from 1.16 to 2.35 per 1000 individuals per year. AT injuries were shown in both sexes but had a higher incidence in middle-aged men [3]. The clinical findings in tendon overuse injuries are a combination of pain, morning stiffness, swelling, and inability to perform sports activities [4]. Li and Hua [5] divided the AT into three categories: Mid-portion AT (located from 2 to 6 cm proximal to the Achilles insertion at the calcaneus); insertional AT (located at the Achilles tendon insertion at the calcaneus) and other disturbances (e.g., bursitis). Boesen et al. [6] argued that overloading plays an essential role in the development and the management of this condition. Achilles load management programs are oriented to work isotonic and eccentrically the lower limb muscles, abdominal wall and paraspinal muscles are involved working in a coordinated manner to provide body stability and balance. In addition, lower limb disturbances, such as AT, could be comprised the transferring load mechanisms through the body, in both directions [7].

Core muscles presented morphological differences and were composed of: Rectus abdominis (RA), external oblique (EO), internal oblique (IO), transversus abdominis (TrAb), and multifidus muscles [8]. Whittaker et al. [9] reported that these muscles worked with the pelvic floor muscles and diaphragm to pressurize the abdominal cavity and to transfer loads through the lower limb and the trunk. Hodges et al. [10] reported functional deficits in individuals with lumbopelvic pain (LPP). In addition, core muscles have shown structural differences in subjects with and without chronic low back pain [11]. Regarding the paraspinal muscles, several authors highlighted the importance of a quantitative and standardized assessment by ultrasound imaging (USI) of the multifidus muscles in order to evaluate the morphology and behavior during contraction and at rest in individuals with and without pathologies [12,13].

Current literature showed evidence about the use of the USI to assess the muscles thickness and cross-sectional area (CSA) as a complement of a physical therapy examination [14,15]. In addition, the evaluations can be performed in static and dynamic B-Mode. Regarding the lower limb muscles, Romero et al. [16] reported a decreasing of the CSA in extensor digitorum longus (EDL), tibialis anterior (TA), and peroneus muscles (PER) in individuals with AT. USI measurements of the abductor hallucis brevis (AHB), and flexor hallucis brevis (FHB) thicknesses, as well as flexor digitorum brevis (FDB) and FHB CSA, were increased in patients with AT with respect to a healthy group [17]. Romero et al. [18] showed a reduced plantar fascia at the calcaneus insertion and also the calcaneal fat pad in subjects with AT. The use of the USI to assess the lower limb has been employed in other populations, such as individuals with ankle sprains and hallux valgus reporting a reduced peroneus longus CSA [19] and a reduced CSA and thickness of the abductor hallucis and flexor hallucis [20], respectively. Considering the trunk region, Whittaker et al. [9] described the relationship between individuals with LPP and changes in abdominal wall muscles. In sports populations USI examination of the abdominal wall muscles and the perimuscular connective tissues (PMCT) between elite and amateur basketball players have been performed. Thickness of the TrAb and PMCT were increased in the left (dominant) side in elite basketball players with respect to amateurs [21,22]. Multifidus ultrasound features were studied in healthy populations [23,24] and subjects with low back pain, reporting a decreasing of the CSA [12]. Different evaluation methods were used to evaluate the core muscles: Magnetic resonance imaging [25,26], electromyography [27,28], and USI. Therefore, several authors suggest that USI provides a complete and a real-time assessment of the muscle and soft tissues features being a valid, non-invasive, and reliable tool [29,30].

For the Achilles tendon, ultrasonography evaluations have focused on tendon morphology in individuals with and without pathology. Prior studies about AT have been assessed detailing structures surrounding the Achilles tendon complex. However, research about muscles and connective tissues involved in the load transferring and biomechanics in subjects with lower limb pathology are still needed. Thus, the aim of the present study was to compare and quantify with USI the inter-recti distance (IRD), the thickness of the RA, EO, IO, TrAb, and multifidus muscles as well as the CSA of the RA and multifidus between individuals with and without chronic mid-portion AT.

## 2. Materials and Methods

### 2.1. Design

The Strengthening the Reporting of Observational Studies in Epidemiology (STROBE) [31] criteria were followed in order to perform this secondary analysis of a cross-sectional study from January to December 2017 [16,17,18]. Previously, the Research and Ethics Committee of La Princesa Hospital (Madrid, Spain) approved the study with the record number 2828A. Before starting this research, the informed consent form was read and signed by all the participants. In addition, the ethical recommendations and considerations of the Helsinki Declaration from 2013 were respected during the course of this study [32].

### 2.2. Sample Size Calculation

The sample size calculation was developed with the G*Power software [33] by the between-groups difference. The IO (mm) variable was employed in a pilot study with 18 subjects (mean ± SD): 9 individuals for the tendinopathy group (7.34 ± 3.21) and 9 individuals for the healthy group (8.63 ± 2.86). A power of 0.80, α error of 0.05 and effect size of 0.42 with 1-tailed hypothesis were used for the sample size calculation. Finally, a sample of 140 subjects was calculated. However, we could recruit for this study a total sample of 143 subjects.

### 2.3. Participants

A sample of 143 participants were recruited and divided into two groups: A group comprised of chronic mid-portion AT (*n* = 71) and group B composed of healthy subjects (*n* = 72). For the AT group, participants were included if they presented: Tendon pain of at least 3 points in visual analogue scale, irritation and dysfunction at 2 to 6 cm from the calcaneal insertion, duration of the symptoms of at least 3 months, and participants under pharmacological or physical interventions during the study course [16]. The exclusion criteria were: Diagnosis of insertional AT [34], fractures, surgeries, ankle sprains, previous interventions with corticosteroids injections in the region of the Achilles tendon, systemic diseases, lower limb, or any disturbances the last 12 months [35], history of Achilles tendon rupture in the affected. The enrollment of the subjects was performed by a specialized medical doctor with more than 10 years of experience in sport medicine and musculoskeletal disorders.

### 2.4. Ultrasonography Imaging Measurements

**Figure 1:**
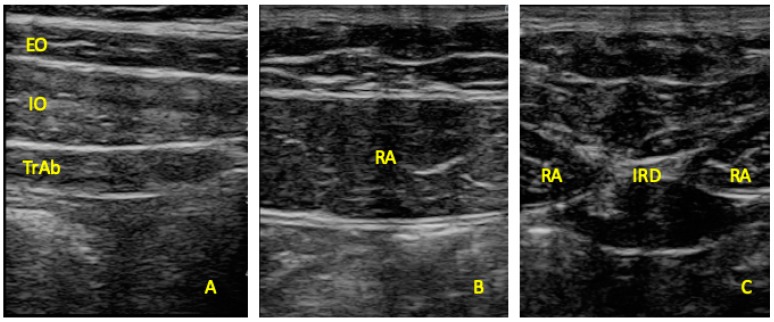
Ultrasound imaging thickness for the EO, IO, TrAb (**A**), RA thickness (**B**) muscles, and IRD (**C**). EO, external oblique; IO, internal oblique; TrAb, transversus abdominis; RA, rectus abdominis; IRD, inter-recti distance.

**Figure 2:**
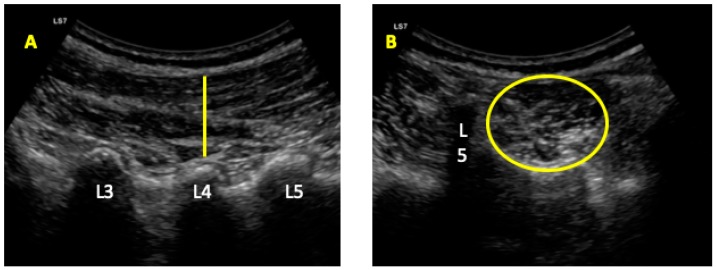
Ultrasound imaging for the multifidus thickness (**A**) and cross-sectional area (CSA) (**B**).

Ultrasonography was developed with a LogiQ ultrasound system (GE, Healthcare, UK), with a 4–13 MHz linear transducer (38-mm footprint) for the anterior abdominal wall muscles and with a 2–5.5 MHz convex transducer (38-mm footprint) for the multifidus evaluations. All the evaluations were developed by the same therapist (P.M.L.), with 3 years of experience in ultrasound imaging of the musculoskeletal field. All the images for the anterior abdominal muscles were taken in supine position. The images of the EO, IO, and TrAb muscles was developed by placing the transductor in the mid-axillary line, between the subcostal line and the iliac crest [9] (Figure 1A). For the RA muscle examination, the transducer was placed aligned with the umbilicus, and just under the umbilicus for the IRD evaluation (Figure 1B). Muscle thickness was considered as the distance between the edges of each muscle and IRD was described as the distance between the both RA muscles [9]. For the multifidus muscles examinations patients were placed lying in prone position at rest and during a maximal isometric contraction with the ipsilateral extended lower limb for 5 s. Following the Wallwork et al. [36] guidelines, the transducer locations were identified by palpations of L4–L5 spinous as the reference points. The thickness of the multifidus muscles was considered as the tip of the target zigapophyseal L4–L5 joint to the inside edge of the superior border of the multifidus muscle [36] (Figure 2A). According to Huang et al. [37], the CSA of the multifidus muscle was recorded with the transducer placed on the skin 25-mm distal from the spinous process of L5 and vertical to the spine (Figure 2B).

Prior studies reported good-to-excellent reliability values for the anterior wall and multifidus muscle USI measurements: EO, IO, and TrAb (0.55–0.97); [38] IRD (0.72–0.91); [39] RA (0.97–0.99); (9) and multifidus thickness (0.84–0.92) [40] and CSA (0.86–0.89) [41]. The mean of 3 repeated values were recorded for each measurement maintaining the transducer at the same place and with the same pressure (just the pressure generated by the weight of the transducer). ImageJ software (Bethesda, MD, USA) was employed to measure all the images offline [42].

### 2.5. Statistics

SPSS software v.26 (IBM SPSS Statistics; NY: IBM) was employed for data analysis. Shapiro–Wilk test was employed to assess the normality [43]. A descriptive analysis was developed for all the subjects and separately in the tendinopathy and health group using mean, standard deviation (SD) to describe the parametric data and mean and interquartile ranges (IR) for non-parametric data. In addition, group differences were assessed employing an independent t-test for parametric data and Mann–Whitney U for non-parametric data.

In order to predict the influence of the descriptive data and group (presence of Achilles tendinopathy) on the statistically significant outcome measurements showed in the prior described analyses, a multivariate analysis was carried out by linear regression. The dependent variables were EO, IO, TrAb, RA, and multifidus thickness as well as IRD and multifidus CSA. The independent variables were group, sex, weight, height, BMI, and age. For all statistical tests, an α error of 0.05 (95% CI) and an β error of 0.2 were employed.

## 3. Results

Considering the Table 1, data analysis showed statistically significant differences in body mass index (BMI) (*p* = 0.12) between the tendinopathy and healthy group. Regarding the Table 2, USI measurements for the EO thickness (*p* = 0.001), IO thickness (*p* = 0.001), TrAb thickness (*p* = 0.041), and RA thickness (*p* = 0.001) were decreased showing statistically significant differences for the tendinopathy group with respect to the healthy group. In addition, the IRD (*p* = 0.001), multifidus thickness (*p* = 0.001), and multifidus CSA (*p* = 0.001) were increasing reporting statistically significant differences for the tendinopathy group with respect the healthy group.

According to the linear regression analysis (Table 3), the prediction model for the IRD (*R*^2^ = 0.494) was determined by weight, height, BMI and group; RA thickness (*R*^2^ = 0.303) was determined by age; EO thickness (*R*^2^ = 0.387) was determined by age, sex and group; IO thickness (*R*^2^ = 0.380) was determined by sex and group; TrAb (*R*^2^ = 0.260) was determined by group; multifidus CSA (*R*^2^ = 0.341) was determined by age and multifidus thickness (*R*^2^ = 0.643) was determined by sex and group.

## 4. Discussion

To the current knowledge, this study may be stated as the first study detailing the abdominal wall muscles and multifidus muscles in patients with chronic mid-portion AT compared with healthy individuals. Our findings were in line with prior research showing a relationship between Achilles Tendinopathy and morphological muscle architecture changes [16,17,18] and regarding the difference of the core muscles between populations [21].

### 4.1. Inter-Recti Distance and Rectus Abdominis Thickness

Prior USI research about abdominal wall muscles have focused on the IO and TrAb muscles [25,44,45]. In addition, Jansen et al. [44] did not found differences in the thickness of the IO and TrAb muscles in LBP compared to healthy individuals. However, our research showed a decrease of the RA thickness and an increased IRD for the tendinopathy group. Coldron et al. [46] reported similar results in postpartum women founding a significantly thinner RA and a wider IRD for the postpartum women. In the same line, Romero et al. [21] compared the abdominal wall muscles by USI between elite basketball players and amateur showing a significantly increase for the IRD in favor the elite basketball group with respect group. These results could be related with the high musculoskeletal system demand those who were exposed the elite basketball players due to the multiple training sessions per week. The RA muscle has the greatest thickness of the abdominal wall muscles [47], playing an important role transferring loads in a coordinated manner with the EO, OI, and TrAb muscles. Mota et al. [48] argued that the exploration by palpation of the IRD is sufficient to the clinical practice. However, the USI is a more accurate method to recommend in future research about IRD. In addition, the work developed by Rankin et al. [47] supports the reliability and validity for the RA muscle examination by USI.

### 4.2. External Oblique, Internal Oblique, and Transversus Abdominis Thickness

Regarding the anterolateral abdominal muscles several authors examined and compared by USI different populations with respect to healthy groups. For example, Jansen et al. [44] showed a smaller TrAb thickness in subjects with longstanding adduction-related groin pain compared with healthy athletes. Rostami et al. [49] compared the thickness of the anterolateral muscles of competitive off-road cyclists with and without LBP showing a lower thickness of the TrAb for the LBP group. In this line, the results of the present investigation presented a decrease of the TrAb, IO, and EO thickness for the tendinopathy group. The important role of the TrAb muscle in control of the spine was widely evidenced, being the deep trunk abdominal muscles considered as main outcomes to assess and manage specific populations with different musculoskeletal disorders such as LPP, LBP, or AT individuals [50,51].

### 4.3. Multifidus Muscles

Wallwork et al. [36] reported the reliability to assess the voluntarily contraction of the multifidus muscles in real-time with USI. In addition, Hides et al. [52] argued that dynamic studies of paraspinal muscles by USI may be useful to provide a visual feedback to be added in a rehabilitation program in populations with pathology, such as LBB or LPP. In this line, the results of the present study showed significant differences in individuals with pathology—chronic mid-portion AT—compared with healthy subjects. However, Hides et al. [52] reported also differences in multifidus muscles in individuals with subacute LBP showing a decreasing of the CSA in favor to the pathology group. Several authors argued that core load management programs increased the multifidus CSA [53], in order to improve the core protection, stabilization in a coordinated manner with the anterior wall muscles.

### 4.4. Clinical Implications

The findings of the present study did not intend to provide a cause or explanation for the AT. Authors suggest that the examination of the core and multifidus muscles could help to carried out a complete diagnosis added to a traditional AT exploration (e.g., symptomatology and manual therapy exploration). Moreover, our results showed that it could be beneficial the implementation of the anterior abdominal and paraspinal muscles approach to a load and manual therapy program for the prevention and management of individuals with AT.

### 4.5. Limitations

The present study was developed in ultrasound B-Mode, not M-Mode or 4-D mode. In addition, color elastrography may be useful to assess the core and paraspinal muscles, which could have been useful for the assessment muscle and soft tissue features. The BMI was calculated with the Quetelet´s formula reporting differences between groups that may influence the results [54]. Thus, the differences founded in the present study could be partially explained due to the Quetelet´s index calculation and according to our linear regression models. Despite of the anterior abdominal wall and multifidus muscles reported a good-to-excellent reliability values, further research will be necessary with the intra and inter-rater ICC of the therapist in the measure of the ultrasound images.

## 5. Conclusions

The thickness of the EO, IO, TrAb, and RA muscles were reduced as well as the IRD, multifidus thickness and multifidus CSA were increased in patients with AT. Consequently, USI abnormalities in the core and paraspinal muscles should be understood within the clinical background in individuals with AT.

## Figures and Tables

**Table 1:** Sociodemographic data, pain scores, and VISA-A scale of the sample.

Data	Tendinopathy (*n* = 71)	Controls (*n* = 70)	*p*-Value Cases vs. Controls
Age, y	45.11 ± 12.75 *	37.61 ± 11.91 *	0.200 **
Weight, kg	76.00 ± 12.00 ^†^	75.00 ± 18.50 ^†^	0.412 ^‡^
Height, m	1.76 ± 0.11 ^†^	1.76 ± 0.12 ^†^	0.566 ^‡^
BMI, kg/m^2^	24.81 ± 2.13 ^†^	23.88 ± 3.67 ^†^	0.012 ^‡^
VAS	2.00 ± 3.00 ^†^	N/A	N/A
VISA-A	56.00 ± 14.00 ^†^	N/A	N/A

Abbreviations: N/A, not applicable; VAS, visual analogue scale. * Mean ± standard deviation (SD) was applied. ** Student´s t-test for independent samples was performed. † Median ± interquartile range (IR) was used. ‡ Mann–Whitney U test was utilized.

**Table 2:** Ultrasound imaging measurements between groups.

Measurement	Tendinopathy (*n* = 71)	Health (*n* = 72)	*p*-Value
IRD (mm)	17.21 ± 8.37 (10.29–22.63) ^†^	9.83 ± 7.75 (2.01–22.05) ^†^	0.001 ^‡^
RA Thickness (mm^2^)	10.19 ± 2.31 (5.21–16.49) *	11.33 ± 2.15 (6.22–21.55) ^†^	0.001 ^‡^
EO Thickness (mm)	3.99 ± 2.30 (2.10–8.71) ^†^	5.56 ± 1.55 (2.63–9.00) *	0.001 ^‡^
IO Thickness (mm)	8.36 ± 3.47 (2.23–15.57) ^†^	11.08 ± 5.04 (4.65–19.50) ^†^	0.001 ^‡^
TrAb Thickness (mm)	3.74 ± 2.43 (13.38–26.89) ^†^	4.70 ± 1.98 (2.35–9.70) ^†^	0.041 ^‡^
Multifidus Thickness (mm)	24.07 ± 5.67 (18.94–31.63) ^†^	12.17 ± 8.48 (8.08–25.13) ^†^	0.001 ^‡^
Multifidus CSA (mm^2^)	1068.58 ± 175.48 (773.79–1610.19) ^†^	960.72 ± 437.52 (665.19–1641.28)	0.001 ^‡^

Abbreviations: CSA, cross sectional area; EO, external oblique; IO, internal oblique; TrAb, transversus abdominis. * Mean ± standard deviation (SD) (minimum–maximum) was applied. † Median ± interquartile range (IR) (minimum–maximum) was used. ‡ Mann–Whitney U test was utilized.

**Table 3:** Multivariate predictive analysis for IRD, EO, and multifidus thickness for patients with Achilles tendinopathy and controls.

Parameter	Model	*P* Value	Model *R*^2^
IRD (mm)	−207.961		0.494
	−1.686 * Weight 112.29 * Height 6.019 * BMI 5.506 * Group	0.006 0.028 0.001 0.001	
RA Thickness (mm)	65.252		0.303
	−1.740 * Age	0.001	
EO Thickness (mm)	19.754		0.387
	−0.025 * Age 1.312 * Sex −1.132 * Group	0.013 0.006 0.001	
IO Thickness (mm)	−29.138		0.380
	4.129 * Sex −2.892 * Group	0.001 0.001	
TrAb (mm)	−15.813		0.260
	−1.061 * Group	0.001	
Multifidus CSA (mm^2^)	−882.850		0.341
	−2.729 * Age	0.048	
Multifidus Thickness (mm)	−103.879		0.643
	−3.653 * Sex 10.116 * Group	0.013 0.001	

Abbreviations: CSA, cross sectional area; EO, external oblique; IO, internal oblique; IRD, interrecti distance; TrAb, transversus abdominis; * Multiplay: Group (control = 0; Tendinopathy = 1); Sex (women = 0; men = 1).

## References

[1] Alfredson H. (2003). Chronic midportion Achilles tendinopathy: An update on research and treatment. Clin. Sports Med..

[2] Albers I.S., Zwerver J., Diercks R.L., Dekker J.H., Van den Akker-Scheek I. (2016). Incidence and prevalence of lower extremity tendinopathy in a Dutch general practice population: A cross sectional study. BMC Musculoskelet. Disord..

[3] Alfredson H., Lorentzon R. (2000). Chronic Achilles tendinosis: Recommendations for treatment and prevention. Sports Med..

[4] Cook J.L., Khan K.M., Purdam C. (2002). Achilles tendinopathy. Man. Ther..

[5] Li H.-Y., Hua Y.-H. (2016). Achilles Tendinopathy: Current Concepts about the Basic Science and Clinical Treatments. Biomed Res. Int..

[6] Boesen A.P., Boesen M.I., Koenig M.J., Bliddal H., Torp-Pedersen S., Langberg H. (2011). Evidence of accumulated stress in Achilles and anterior knee tendons in elite badminton players. Knee Surg. Sports Traumatol. Arthrosc..

[7] Richardson C.A., Snijders C.J., Hides J.A., Damen L., Pas M.S., Storm J. (2002). The relation between the transversus abdominis muscles, sacroiliac joint mechanics, and low back pain. Spine.

[8] Brown S.H.M., Ward S.R., Cook M.S., Lieber R.L. (2011). Architectural analysis of human abdominal wall muscles: Implications for mechanical function. Spine.

[9] Whittaker J.L., Warner M.B., Stokes M. (2013). Comparison of the Sonographic Features of the Abdominal Wall Muscles and Connective Tissues in Individuals With and Without Lumbopelvic Pain. J. Orthop. Sports Phys. Ther..

[10] Hodges P.W., Moseley G.L. (2003). Pain and motor control of the lumbopelvic region: Effect and possible mechanisms. J. Electromyogr. Kinesiol..

[11] Radebold A., Cholewicki J., Panjabi M.M., Patel T.C. (2000). Muscle response pattern to sudden trunk loading in healthy individuals and in patients with chronic low back pain. Spine.

[12] Hides J., Gilmore C., Stanton W., Bohlscheid E. (2008). Multifidus size and symmetry among chronic LBP and healthy asymptomatic subjects. Man. Ther..

[13] Stokes M., Hides J., Elliott J., Kiesel K., Hodges P. (2007). Rehabilitative ultrasound imaging of the posterior paraspinal muscles. J. Orthop. Sports Phys. Ther..

[14] Chang K.-V., Yang K.-C., Wu W.-T., Huang K.-C., Han D.-S. (2019). Association between metabolic syndrome and limb muscle quantity and quality in older adults: A pilot ultrasound study. Diabetes Metab. Syndr. Obes. Targets Ther..

[15] Chang K.-V., Wu W.-T., Huang K.-C., Jan W.H., Han D.-S. (2018). Limb muscle quality and quantity in elderly adults with dynapenia but not sarcopenia: An ultrasound imaging study. Exp. Gerontol..

[16] Romero-Morales C., Martin-Llantino P.J., Calvo-Lobo C., Sanchez-Gomez R., Lopez-Lopez D., Pareja-Galeano H., Rodriguez-Sanz D. (2019). Ultrasound evaluation of extrinsic foot muscles in patients with chronic non-insertional Achilles tendinopathy: A case-control study. Phys. Ther. Sport.

[17] Romero-Morales C., Martin-Llantino P.J., Calvo-Lobo C., Almazan-Polo J., Lopez-Lopez D., de la Cruz-Torres B., Palomo-Lopez P., Rodriguez-Sanz D. (2019). Intrinsic foot muscles morphological modifications in patients with Achilles tendinopathy: A novel case-control research study. Phys. Ther. Sport.

[18] Romero-Morales C., Martín-Llantino P.J., Calvo-Lobo C., López-López D., Sánchez-Gómez R., De-La-Cruz-Torres B., Rodríguez-Sanz D. (2019). Ultrasonography Features of the Plantar Fascia Complex in Patients with Chronic Non-Insertional Achilles Tendinopathy: A Case-Control Study. Sensors.

[19] Lobo C.C., Morales C.R., Sanz D.R., Corbalan I.S., Marin A.G., Lopez D.L. (2016). Ultrasonography Comparison of Peroneus Muscle Cross-sectional Area in Subjects With or Without Lateral Ankle Sprains. J. Manip. Physiol. Ther..

[20] Lobo C.C., Marin A.G., Sanz D.R., Lopez D.L., Lopez P.P., Morales C.R., Corbalan I.S. (2016). Ultrasound evaluation of intrinsic plantar muscles and fascia in hallux valgus: A case-control study. Medicine.

[21] Romero-Morales C., Almazán-Polo J., Rodríguez-Sanz D., Palomo-López P., López-López D., Vázquez-González S., Calvo-Lobo C. (2018). Rehabilitative Ultrasound Imaging Features of the Abdominal Wall Muscles in Elite and Amateur Basketball Players. Appl. Sci..

[22] Morales C.R., Polo J.A., Sanz D.R., Lopez D.L., Gonzalez S.V., Buria J.L.A., Lobo C.C. (2018). Ultrasonography features of abdominal perimuscular connective tissue in elite and amateur basketball players: An observational study. Rev. Assoc. Med. Bras..

[23] Shi J., Zheng Y.P., Chen X., Huang Q.H. (2007). Assessment of muscle fatigue using sonomyography: Muscle thickness change detected from ultrasound images. Med. Eng. Phys..

[24] Chang K.-V., Kara M., Su D.C.-J., Gurcay E., Kaymak B., Wu W.-T., Ozcakar L. (2019). Sonoanatomy of the spine: A comprehensive scanning protocol from cervical to sacral region. Med. Ultrason..

[25] Hides J.A., Belavy D.L., Stanton W., Wilson S.J., Rittweger J., Felsenberg D., Richardson C.A. (2007). Magnetic resonance imaging assessment of trunk muscles during prolonged bed rest. Spine.

[26] Hides J.A., Boughen C.L., Stanton W.R., Strudwick M.W., Wilson S.J. (2010). A magnetic resonance imaging investigation of the transversus abdominis muscle during drawing-in of the abdominal wall in elite Australian Football League players with and without low back pain. J. Orthop. Sports Phys. Ther..

[27] Kiesel K.B., Uhl T.L., Underwood F.B., Rodd D.W., Nitz A.J. (2007). Measurement of lumbar multifidus muscle contraction with rehabilitative ultrasound imaging. Man. Ther..

[28] Kim J.-S., Kang M.-H., Jang J.-H., Oh J.-S. (2015). Comparison of selective electromyographic activity of the superficial lumbar multifidus between prone trunk extension and four-point kneeling arm and leg lift exercises. J. Phys. Ther. Sci..

[29] Teyhen D.S., Gill N.W., Whittaker J.L., Henry S.M., Hides J.A., Hodges P. (2007). Rehabilitative ultrasound imaging of the abdominal muscles. J. Orthop. Sports Phys. Ther..

[30] Romero-Morales C., Martin-Llantino P.J., Calvo-Lobo C., Palomo-Lopez P., Lopez-Lopez D., Pareja-Galeano H., Rodriguez-Sanz D. (2019). Comparison of the sonographic features of the Achilles Tendon complex in patients with and without achilles tendinopathy: A case-control study. Phys. Ther. Sport.

[31] von Elm E., Altman D.G., Egger M., Pocock S.J., Gotzsche P.C., Vandenbroucke J.P. (2014). The Strengthening the Reporting of Observational Studies in Epidemiology (STROBE) Statement: Guidelines for reporting observational studies. Int. J. Surg..

[32] Holt G.R. (2014). Declaration of Helsinki-the world’s document of conscience and responsibility. South. Med. J..

[33] Faul F., Erdfelder E., Lang A.-G., Buchner A. (2007). G * Power 3: A flexible statistical power analysis program for the social, behavioral, and biomedical sciences. Behav. Res. Methods.

[34] Habets B., van Cingel R.E.H., Backx F.J.G., Huisstede B.M.A. (2017). Alfredson versus Silbernagel exercise therapy in chronic midportion Achilles tendinopathy: Study protocol for a randomized controlled trial. BMC Musculoskelet. Disord..

[35] Alfredson H., Cook J. (2007). A treatment algorithm for managing Achilles tendinopathy: New treatment options. Br. J. Sports Med..

[36] Wallwork T.L., Hides J.A., Stanton W.R. (2007). Intrarater and interrater reliability of assessment of lumbar multifidus muscle thickness using rehabilitative ultrasound imaging. J. Orthop. Sports Phys. Ther..

[37] Huang Q., Li D., Zhang Y., Hu A., Huo M., Maruyama H. (2014). The Reliability of Rehabilitative Ultrasound Imaging of the Cross-sectional Area of the Lumbar Multifidus Muscles in the PNF Pattern. J. Phys. Ther. Sci..

[38] Park S. (2013). doo Reliability of Ultrasound Imaging of the Transversus Deep Abdominial, Internal Oblique and External Oblique Muscles of Patients with Low Back Pain Performing the Drawing-in Maneuver. J. Phys. Ther. Sci..

[39] Keshwani N., Hills N., McLean L. (2016). Inter-Rectus Distance Measurement Using Ultrasound Imaging: Does the Rater Matter?. Physiother. Can..

[40] Sions J.M., Velasco T.O., Teyhen D.S., Hicks G.E. (2014). Ultrasound imaging: Intraexaminer and interexaminer reliability for multifidus muscle thickness assessment in adults aged 60 to 85 years versus younger adults. J. Orthop. Sports Phys. Ther..

[41] Nabavi N., Mosallanezhad Z., Haghighatkhah H.R., Mohseni Bandpeid M.A. (2014). Reliability of rehabilitative ultrasonography to measure transverse abdominis and multifidus muscle dimensions. Iran. J. Radiol..

[42] Schneider C.A., Rasband W.S., Eliceiri K.W. (2012). NIH Image to ImageJ: 25 years of image analysis. Nat. Methods.

[43] Ghasemi A., Zahediasl S. (2012). Normality tests for statistical analysis: A guide for non-statisticians. Int. J. Endocrinol. Metab..

[44] Jansen J., Weir A., Denis R., Mens J., Backx F., Stam H. (2010). Resting thickness of transversus abdominis is decreased in athletes with longstanding adduction-related groin pain. Man. Ther..

[45] Stuge B., Morkved S., Dahl H.H., Vollestad N. (2006). Abdominal and pelvic floor muscle function in women with and without long lasting pelvic girdle pain. Man. Ther..

[46] Coldron Y., Stokes M.J., Newham D.J., Cook K. (2008). Postpartum characteristics of rectus abdominis on ultrasound imaging. Man. Ther..

[47] Rankin G., Stokes M., Newham D.J. (2006). Abdominal muscle size and symmetry in normal subjects. Muscle Nerve.

[48] Mota P., Pascoal A.G., Sancho F., Carita A.I., Bo K. (2013). Reliability of the inter-rectus distance measured by palpation. Comparison of palpation and ultrasound measurements. Man. Ther..

[49] Rostami M., Ansari M., Noormohammadpour P., Mansournia M.A., Kordi R. (2015). Ultrasound assessment of trunk muscles and back flexibility, strength and endurance in off-road cyclists with and without low back pain. J. Back Musculoskelet. Rehabil..

[50] Sutherlin M.A., Gage M., Mangum L.C., Hertel J., Russell S., Saliba S.A., Hart J.M. (2018). Changes in Muscle Thickness Across Positions on Ultrasound Imaging in Participants With or Without a History of Low Back Pain. J. Athl. Train..

[51] Whittaker J.L. (2008). Ultrasound imaging of the lateral abdominal wall muscles in individuals with lumbopelvic pain and signs of concurrent hypocapnia. Man. Ther..

[52] Hides J.A., Saide M., Stokes M.J., Jull G.A., Cooper D.H. (1994). Evidence of lumbar multifidus muscle wasting ipsilateral to symptoms in patients with acute/subacute low back pain. Spine.

[53] Kliziene I., Sipaviciene S., Klizas S., Imbrasiene D. (2015). Effects of core stability exercises on multifidus muscles in healthy women and women with chronic low-back pain. J. Back Musculoskelet. Rehabil..

[54] Garrow J.S., Webster J. (1985). Quetelet’s index (W/H2) as a measure of fatness. Int. J. Obes..

